# The Japanese Breast Cancer Society clinical practice guidelines for epidemiology and prevention of breast cancer, 2022 edition

**DOI:** 10.1007/s12282-023-01531-9

**Published:** 2023-12-26

**Authors:** Masaaki Kawai, Shoichiro Ohtani, Motoki Iwasaki, Seiichiro Yamamoto, Kiyoshi Takamatsu, Hitoshi Okamura, Masami Arai, Tsunehisa Nomura, Shinji Ozaki, Ken-ichi Shibata, Ayaka Akabane, Fuyuhiko Motoi, Chikako Yamauchi, Yutaka Yamamoto, Hiroji Iwata, Shigehira Saji

**Affiliations:** 1 Department of Surgery I, Yamagata University, Yamagata, Japan; 2 Ohtani Shoichiro Breast Clinic, Hiroshima, Japan; 3 Division of Epidemiology, National Cancer Center Institute for Cancer Control, Tokyo, Japan; 4 Shizuoka Graduate University of Public Health, Shizuoka, Japan; 5 Department of Obstetrics and Gynecology, Tokyo Dental College Ichikawa General Hospital, Ichikawa, Japan; 6 Department of Psychosocial Rehabilitation, Graduate School of Biomedical and Health Sciences, Hiroshima University, Higashi-Hiroshima, Japan; 7 Department of Clinical Genetics, Juntendo University, Graduate School of Medicine, Tokyo, Japan; 8 Department of Breast and Thyroid Surgery, Kawasaki Medical School, Matsushima, Kurashiki Japan; 9 Department of Breast Surgery, Hiroshima Prefectural Hospital, Hiroshima, Japan; 10 Department of Radiation Oncology, Shiga General Hospital, Moriyama, Moriyama-Shi, Shiga Japan; 11 Department of Breast and Endocrine Surgery, Kumamoto University Hospital, Kumamoto, Japan; 12 Department of Breast Oncology, Aichi Cancer Center Hospital, Nagoya, Japan; 13 Department of Medical Oncology, Fukushima Medical University, Fukushima, Japan

**Keywords:** Epidemiology, Prevention, Breast, Guideline, JBCS, 2022

## Abstract

The Japanese Breast Cancer Society Clinical Practice Guidelines for Epidemiology and Prevention of Breast Cancer, 2022 Edition.

## Introduction

The evidence-based Clinical Practice Guidelines published by the Japanese Breast Cancer Society (JBCS) define important themes in daily clinical practice as clinical questions (CQs). Each CQ is answered through a comprehensive literature search, preparation of a narrative based on a critical review of the literature, a recommendation after a review by committee members, and finalizing of this recommendation. CQs with positive results in randomized controlled trials (RCTs) or meta-analyses are typically "strongly recommended" as having Level 1 evidence. The 2018 [1] and later editions of the guidelines are designed to provide both physicians and patients with support tools to utilize shared decision making. These editions were developed in compliance with the Minds (Medical Information Distribution Service) Manual for Clinical Practice Guideline Development 2020 ver. 3.0 where possible [2]. The JBCS Clinical Practice Guidelines for Breast Cancer, 2022 edition were published in Japanese in June 2022 [3, 4].

The first edition of the JBCS Clinical Practice Guidelines for Epidemiology was published in 2004 and 2005. After revision every 3 years, these guidelines were published in 2011 as the JBCS Clinical Practice Guidelines for Epidemiology and Prevention of Breast Cancer. Revised editions were published in 2013 [5] and 2015 [6], with revisions made every 2 years to keep up with the rapid accumulation of data and changes in the standard of care. A fully revised edition was published in 2018. Here, we provide a summary of the recent 2022 edition of the JBCS Clinical Practice Guidelines for Epidemiology and Prevention of Breast Cancer, which we believe will provide useful information for patients and physicians in Japan and overseas [3, 4].

## Structure of the JBCS clinical practice guidelines for breast cancer, 2022 edition

Review: This section describes the basic concepts, flow of treatment, definitions of terms, historical course, and minimum necessary textbook knowledge.

Background Question (BQ): These questions address issues positioned as standard treatment and viewed as a practice that must be implemented, or those that are widely implemented, but for which no new data have emerged to strengthen the rationale.

Clinical Question (CQ): These questions consider a topic that is difficult to judge in daily clinical practice. A quantitative or qualitative systematic review is conducted. The strength of evidence (SoE) is indicated in the recommendation narrative. The recommendation itself and the strength of recommendation (SoR) are determined through a vote at the recommendation decision-making meeting, and an explanation is provided based on the discussion at this meeting.

The SoRs for CQs are shown in Table 1. The SoR was determined based on the balance of risks and benefits of the intervention in daily clinical practice, consistency with patient preferences, and economic perspectives. The SoR is divided into four grades according to the Minds Manual for Clinical Practice Guideline Development 2020 ver. 3.0. [2] The SoE is indicated in the recommendation narrative at one of four levels: "Strong", "Moderate", "Weak" and "Very weak" (Table 2). For all outcomes for each CQ, the stronger the overall evidence, the stronger the recommendation tends to be. However, in some cases, even if the SoE is "Moderate", the SoR may still be “Strongly recommended to do”, while in other cases, even if the SoE is "Strong", the SoR is “Weakly recommended to do”.

**Table 1:** Strength of recommendation (SoR)

Strength of recommendation	Statement	Clinical meaning
1	Strongly recommend to do	Strongly recommended to be performed
2	Weakly recommend to do	Not that it must be done, but rather that it should be done after consultation on site, based on the balance of benefits and harms and the patient's values, etc
3	Weakly recommend not to do	The opposite of the weak recommendation is that it should not be performed based on the balance of benefits and harms and the patient's values
4	Strongly recommend not to do	Intervention with harms substantially outweighing benefits and strongly recommended not to be performed

**Table 2:** Strength of evidence (SoE) for overall outcomes for recommendation decisions

Strong	Strong confidence in the adequacy of the effect to support the recommendation
Moderate	Moderate confidence in the adequacy of the effect to support the recommendation
Weak	Weak confidence in the adequacy of the effect to support the recommendation
Very weak	Very weak confidence in the adequacy of the effect to support the recommendation

Most epidemiology CQs do not address interventions, but rather consider issues to be aware of in daily life. Therefore, except for CQ3, CQ4, CQ5 and CQ8, we specify the certainty of the scientific basis as an evidence grade based on "Food, Nutrition, Physical Activity, and the Prevention of Cancer: A Global Perspective, 2nd Edition (2007)" [7] published by the World Cancer Research Fund (WCRF)/American Institute for Cancer Research (AICR) (https://wcrf.org/) (Table 3). The second edition of the guidelines evaluates the certainty of a causal relationship based on a review of the evidence, using the categories: "Convincing", "Probable", "Limited-suggestive", "Limited-no conclusion" and "Substantial effect on risk unlikely”. To be considered "Convincing", there must be evidence from multiple cohort studies, the results must be consistent, the study must be of high quality with as little bias as possible, there must be a dose–response relationship, and the biological mechanism must be explained by animal experiments or other means. The evaluation "Probable" requires evidence from multiple cohort studies and fulfillment of requirements other than the dose–response relationship. Thus, preventive actions are recommended for factors rated "Convincing" or "Probable". "Limited-suggestive" is applicable when there is evidence from multiple cohort studies, the results of which are generally consistent, and the biological mechanism can be explained by data from animal experiments, etc., although the methodology of the studies may be problematic or the number of studies may be small. An evaluation of "Limited-no conclusion" is made when a more definitive evaluation cannot be made due to the small number of studies, inconsistent results, or low quality of the studies. Therefore, preventive actions are not recommended for factors rated "Limited-suggestive" or "Limited-no conclusion". A rating of “Substantial effect on risk unlikely" is given when there is evidence from multiple cohort studies, the lowest and highest intake groups are consistently found to have a risk close to 1, and there are high quality studies that eliminate as much bias as possible.

**Table 3:** Evidence grade (only used in the area of epidemiology and prevention)

Convincing	There is sufficient evidence to conclude with certainty that there is an association with cancer risk, and preventive actions are recommended
Probable	There is sufficient evidence to conclude with near certainty that there is an association with cancer risk, and preventive actions are generally recommended
Limited-suggestive	Cannot be judged as "Convincing" or "Probable," but there is evidence to suggest an association with cancer risk
Limited-no conclusion	Insufficient data to draw conclusions about the association with cancer risk
Substantial effect on risk unlikely	There is sufficient evidence to conclude that there is no substantial effect on the risk of carcinogenesis

Evidence grade was determined based on “Food, Nutrition, Physical Activity, and the Prevention of Cancer: A Global Perspective, 2nd Edition (2007)” published by the World Cancer Research Fund (WCRF)/American Institute for Cancer Research (AICR) (https://wcrf.org/) [7]

The percentage consensus (%) at the recommendation meeting is given to improve the reader’s understanding of the recommendation statement based on some experts giving a strong recommendation, whereas others may have weakly opposed the recommendation. It is also possible to understand whether there is a difference of opinion based on the decision being made by a single vote or multiple votes. In other words, in shared decision making, it is important to disclose that there are differences of opinion among experts and to use this information for decision making with patients. Each CQ and recommendation also has a “Key points” section, which gives the conditions, information, and points to note for understanding the recommendation.

## Descriptive epidemiology of breast cancer in Japan

1. Mortality

**Fig. 1:**
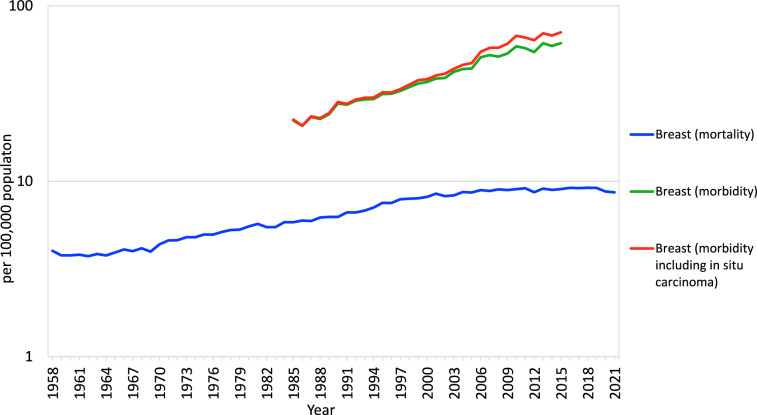
Annual trends in breast cancer morbidity and mortality among Japanese women by age standardization on the world population (per 100,000 population). From: Updated trends in cancer in Japan: incidence in 1985–2015 and mortality in 1958–2018 a sign of decrease in cancer incidence. J Epidemiol. 2021; 31: 426–450 [8]. Cancer Statistics. Cancer Information Service, National Cancer Center, Japan (Vital Statistics of Japan, Ministry of Health, Labour and Welfare) (1958–2019)

All cancer deaths in Japan are recorded in the Current Population Survey of the Ministry of Health, Labour and Welfare (MHLW). Cancer mortality data from the Vital Statistics (1958–2019) and graphs based on these data are available from the National Cancer Center Cancer Information Service, "Cancer Statistics" (https://ganjoho.jp/reg_stat/index.html). In the 2021 Vital Statistics, the number of cancer deaths in women was 159,038, of which breast cancer accounted for 14,803 (9.3% of all cancer deaths). Breast cancer mortality ranked fifth after colon (24,338), lung (22,934), pancreas (19,245), and stomach (18,590) cancer, and age-adjusted mortality has shown an increasing trend since the 1960s (Fig. 1).

**Fig. 2:**
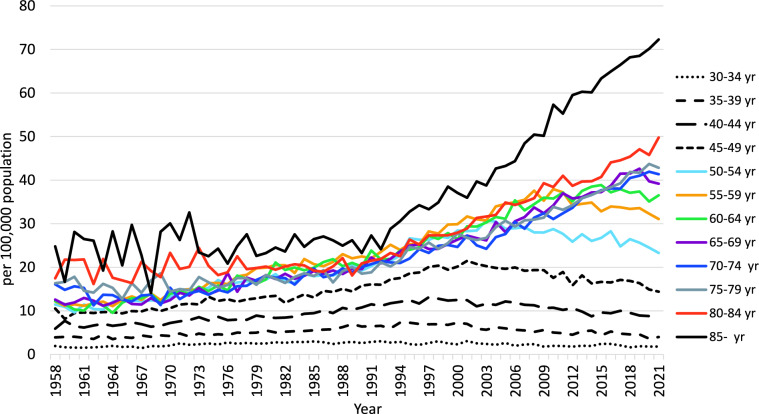
Annual trends in breast cancer mortality among Japanese women by age group (per 100,000 population). From: Cancer Statistics. Cancer Information Service, National Cancer Center, Japan (Vital Statistics of Japan, Ministry of Health, Labour and Welfare) (1958–2019)

**Fig. 3:**
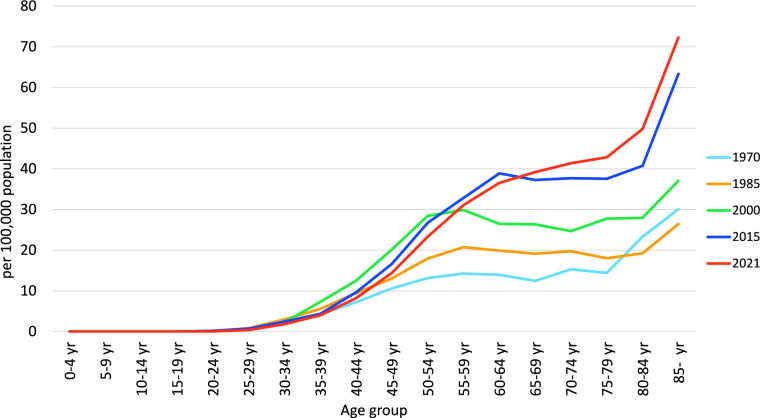
Breast cancer mortality among Japanese women by age group (per 100,000 population). From: Cancer Statistics. Cancer Information Service, National Cancer Center, Japan (Vital Statistics of Japan, Ministry of Health, Labour and Welfare) (Year 1958–2019)

Annual trends in breast cancer mortality by age group (Fig. 2) show that increases in recent years are more pronounced in patients aged ≥ 60 years old. In contrast, there has been a decreasing trend in the 40–54 age group since around 2000. The decrease in mortality in this age group may have contributed to the slowing of the increase in breast cancer mortality in Japanese women. The mortality by age group (Fig. 3) shows that in 1970, 1985 and 2000 mortality peaked in the 55–59 age group and then leveled off with age, but increased in the 80 s age group. Similarly, in 2015, mortality peaked in the 60–64 age group and then leveled off with age, but also increased in the 80 s age group. The peak mortality rates in 2015 and 2021 are in older age groups than in 2000, and mortality is lower in the 40 to early 50 s age group, reflecting the recent decline in mortality at this age.

(2) Morbidity

Unlike for mortality, a national system for collection of data on cancer incidence has not been established in Japan. In December 2013, the Act on Promotion of a Cancer Registry was enacted, and a national cancer registry was launched in January 2016. In national cancer registry data in 2018, the number of cases in women was 421,964, of which 93,858 were breast cancer. This represents 22.2% of all cancers and is the most frequent site of cancer in women. The incidence data shown in Fig. 1 were compiled by combining data from the regional cancer registries in Yamagata, Fukui, and Nagasaki prefectures for the purpose of examining annual trends. The cancer registries in these three prefectures have been accurate and stable over the long term, and changes in the accuracy of the registries are thought to have little effect on the high and low morbidity rates.

**Fig. 4:**
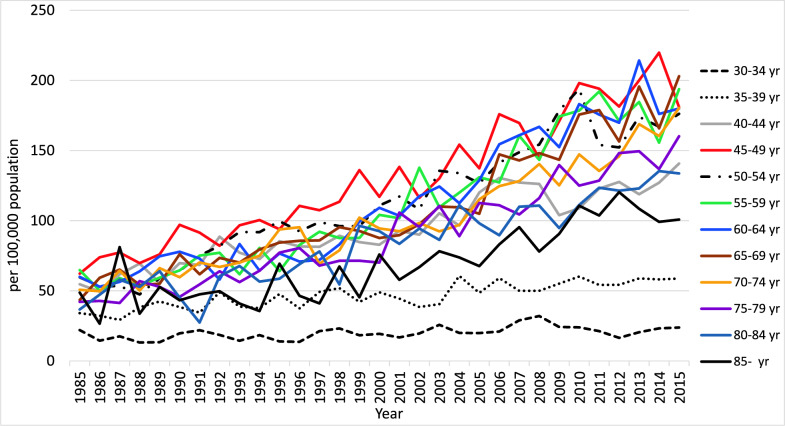
Yearly change in breast cancer morbidity among Japanese women by age group (per 100,000 population). From: Updated trends in cancer in Japan: incidence in 1985–2015 and mortality in 1958–2018—a sign of decrease in cancer incidence. J Epidemiol. 2021; 31: 426–450 [8]

**Fig. 5:**
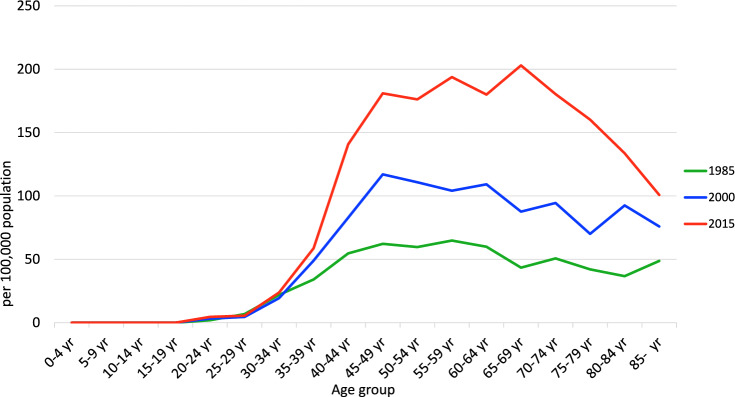
Breast cancer incidence among Japanese women by age group (per 100,000 population). From: Updated trends in cancer in Japan: incidence in 1985–2015 and mortality in 1958–2018 a sign of decrease in cancer incidence. J Epidemiol. 2021; 31: 426–450 [8]

Annual trends in breast cancer incidence by age group (Fig. 4) show a high incidence in the 45–54 age group, although annual trends vary greatly from year to year. The incidence in the 45 and older age group generally shows an increasing trend [8]. The incidence by age group (Fig. 5) shows a rapid increase until age 45, a peak between 45 and 69, and then leveling off or a gradual decrease, although the rate has varied from year to year [8].

(3) International variation in breast cancer incidence

Information on global cancer incidence is published by the International Agency for Research on Cancer as the "Cancer Incidence in Five Continents" based on information from regional cancer registries in each country [9]. For mortality, the World Health Organization (WHO) compiles mortality data by cause of death for each country in its Mortality Database. The age-adjusted incidence rate of breast cancer among Japanese women is about one-half that of Western countries, and annual trends show that the rate in Japan has leveled off in recent years, while the rate in China has continued to increase. The age-adjusted mortality of breast cancer among Japanese women is about two-thirds that of Western countries. The annual mortality rate in Western countries began to decline around 1990, whereas in Japan, the increase has recently slowed down.

## Risk factors for breast cancer

1. Association of physiological and reproductive factors with risk for breast cancer

A pooled analysis of 9 cohort studies of 187,999 Japanese women (premenopausal 61,113; postmenopausal 126,886) [10] showed no significant association of age at menarche and lactation history with risk for breast cancer for pre- or postmenopausal women. The risk was significantly higher in women with age at menopause ≥ 50 years compared with ≤ 44 years. There was a significant risk reduction in premenopausal women with two or more births compared to those with no births, and in postmenopausal women as the number of births increased. The risk was also significantly higher in premenopausal women with age at first childbirth ≥ 36 years compared to 21–25 years, and in postmenopausal women with age at first childbirth ≥ 26 years.

Although this pooled analysis is the largest among studies of Japanese women, it is noteworthy that the numbers of cases of breast cancer were only 873 in premenopausal women and 1,456 in postmenopausal women; that some of the cohort studies assessed age at menarche, menopause, and first childbirth using a choice of categories and thus, could not calculate the risk per year; and that some studies did not include a risk per year of age at menarche, menopause, or first childbirth. In addition, some studies did not collect information on lactation period, and the association between this period and risk could not be evaluated. These are limitations in evidence for risk in Japanese women, but large international studies also suggest that age at menarche, age at menopause, childbearing history, age at first childbirth, and lactation history are associated with the risk of developing breast cancer.

(2) Association between diet-related factors and risk for breast cancer

The relationship between breast cancer and food/nutrition has been widely studied mainly in Western countries, and considerable evidence has been accumulated. The WCRF/AICR report, "Diet, Nutrition, Physical Activity, and Cancer: A Global Perspective” is a representative evaluation of causal relationships based on this evidence. Since the first edition was published in 1997, this report has been widely used by governments, medical professionals, and researchers worldwide as reliable evidence based on uniform standards. In November 2007, the second edition was published to update the evidence [11], and in 2018, the third edition became available as a series of site-specific updates. Here, we review the international status of causality assessment of the association of diet-related factors with the risk for breast cancer, based on the most recent WCRF/AICR report.

Because risk factors may differ between breast cancer diagnosed before and after menopause, the evaluation was divided into pre- and postmenopausal periods. In both periods, adult taller height was rated as a "Convincing" risk factor and breastfeeding as a "Probable" protective factor. Alcohol was also a risk factor for a pre- or postmenopausal diagnosis, but was rated "Probable" before menopause and "Convincing" after menopause. Obesity (including abdominal obesity) was rated as a "Convincing" risk factor in the postmenopausal period, but "Probable" in the premenopausal period. Postmenopause, weight gain in adulthood was rated as a "Convincing" risk factor, while obesity in adolescence and adulthood from ages 18 to 30 was a "Probable" protective factor. Physical activity was a protective factor before and after menopause, but was rated "Limited-suggestive" before menopause and "Probable" after menopause. Particularly intense physical activity was rated "Probable" before menopause. Heavy birth weight before menopause was another "Probable" risk factor. In pre- and postmenopausal women, non-starchy vegetables (estrogen receptor-negative only), carotenoids in food, and a diet high in calcium were "Limited-suggestive" protective factors. Consumption of dairy products was also a "Limited-suggestive" protective factor in premenopausal women. No other factors were classified as "Limited-suggestive" or higher. Many foods and nutrients were rated as "Limited-no conclusion”, but none were judged as "Substantial effect on risk unlikely”. Alcohol, obesity, physical activity, dairy products, soy and soy products, coffee, and isoflavones are addressed in BQs later in this article. Details are given in the respective sections.

The current status of evidence-based assessments for Japanese people is presented in Table 4. These are results from the "Development and Evaluation of Cancer Prevention Strategies in Japan" research group funded by the National Cancer Center Institute for Cancer Control (https://epi.ncc.go.jp/en/can_prev/index.html). This group also established criteria based on the methods of the WCRF and other international organizations, and conducted a causal relationship evaluation based on evidence in the Japanese population. Postmenopausal obesity was rated as a "Convincing" risk factor, and premenopausal obesity (BMI > 30 kg/m^2^), smoking, passive smoking, and alcohol intake as "Limited-suggestive" risk factors. The postmenopausal obesity risk was consistent with the international assessment, whereas the premenopausal obesity risk was the opposite of this assessment. In the Japanese data, exercise, breastfeeding, soy and isoflavone intake were rated as "Limited-suggestive" protective factors. This rating is lower than that for exercise and breastfeeding as protective factors in the international evaluation, but higher than the rating of "Limited-no conclusion" for soy and isoflavone intake in the international evaluation.

**Table 4:** Evaluation of the association between lifestyle factors and breast cancer in the Japanese population

Evidence grade	Risk factor	Protective factor
Convincing	Obesity (postmenopausal)	
Probable		
Limited-suggestive	Smoking, Passive smoking, Alcohol consumption, Obesity (premenopausal, BMI > 30)	Exercise, Lactation, Soy, Isoflavone
Limited-no conclusion	Vegetables, Fruits, Meat, Fish, Grains, Milk and dairy products, Food patterns, Green tea, Folic acid, Vitamins, Carotenoids, Fats	

### Hereditary breast cancer, genetic testing, and genetic counseling

It is believed that 5–10% of breast cancers are hereditary; i.e., patients carry germline mutations of genes related to development of breast cancer. From a secondary prevention perspective, it is important for the genetic risk for breast cancer to be evaluated and early medical intervention provided for those at high risk to improve their prognosis. In Japan, the medical reimbursement revision in April 2020 allows for some *BRCA1/2* gene tests and additional genetic counseling, breast MRI for surveillance of *BRCA* pathological variant carriers under certain conditions, and risk-reducing mastectomy (RRM) and risk-reducing surgery for oophorectomy (RRSO) covered by insurance.

There is increased awareness of hereditary breast and ovarian cancer syndrome (HBOC) among medical professionals, and it has become clear that a relatively high number of Japanese patients have hereditary breast cancer with pathological variants in *BRCA1* or *BRCA2*. [12] A nationwide registry of *BRCA* genetic test examinees has begun, and the clinical and genetic characteristics of *BRCA1* and *BRCA2* pathological variant carriers are starting to be clarified. [13] The Japanese Society for Genetic Counseling and the Japanese Society of Human Genetics jointly established the Certified Genetic Counselor system, which is gradually being implemented in medical practice (356 counselors, as of April, 2023). However, the history of genetic counseling for cancer in Japan is still young, and it was not until the latter half of the 1990s that it became a full-fledged effort.

In June 2018, *BRCA* genetic testing was covered as a companion diagnostic to PARP inhibitors for "*BRCA* mutation-positive and HER2-negative, inoperable or recurrent breast cancer with a history of cancer chemotherapy". From April 2020, *BRCA* genetic testing is covered for patients with breast cancer who meet one of the following conditions:

- Breast cancer with onset at age 45 or younger
- Breast cancer with onset at age 60 or younger and triple-negative subtype
- Two or more primary breast cancers on both sides or one side
- Male breast cancer
- Complicated by ovarian, fallopian tube, or peritoneal cancer at the time of breast cancer diagnosis
- Family history of breast, ovarian, or pancreatic cancer in blood relatives (within the third degree of consanguinity)

Contralateral RRM and breast reconstruction surgery, and RRSO are also now covered as risk-reducing surgeries for patients with breast cancer with a *BRCA* pathological variant. Surveillance of the affected breast after breast-conserving therapy, the contralateral breast without CRRM, and the ovary without RRSO are also covered if the patient does not undergo risk-reducing surgery.

In August 2022, PARP inhibitors were approved for the indication of "postoperative pharmacological treatment of *BRCA* mutation-positive, HER2-negative breast cancer at high risk of recurrence," and *BRCA* genetic testing as a companion diagnostic was additionally covered by insurance. However, similar testing for unaffected relatives with cancer and surveillance and risk-reducing surgery for unaffected *BRCA* pathological variant carriers are still not covered by insurance.

## Relationship between lifestyle and breast cancer prognosis

The WCRF/AICR report [7] is an important source of evidence for the effects of nutrition on risk for breast cancer. Data for diet, nutrition, physical activity and breast cancer survivors were published in 2014 as part of a continuous update of this report [11]. It is important to examine the impact of lifestyle on the ability of women with breast cancer to both survive the disease and live longer. There is still much to be clarified in this relatively new field, but some evidence has emerged that lifestyle may improve prognosis and reduce the risk of second cancers. This evidence is summarized below.

Associations of diet, weight, and physical activity with breast cancer mortality, secondary breast cancer, and other diseases were examined in women with breast cancer, including those who had already been cured. A total of 85 studies including 164,416 patients and 42,572 deaths were reviewed. It was concluded that the quality and quantity of the studies were insufficient and that the evidence was not strong enough to make specific recommendations for breast cancer prognosis. However, several factors were suggested to be associated with prognosis: "healthy weight," "being active," "eating fiber-containing foods," "eating soy-containing foods," and "reducing total fat intake", especially saturated fat intake. This guideline addresses CQs for obesity (CQ6), fat intake (CQ7), physical activity (CQ8), alcohol intake (CQ9) [14], smoking (CQ10), isoflavones (CQ11), and dairy products (CQ12).

The overall recommendation is that post-treatment advice for breast cancer should follow cancer prevention guidelines unless these conflict with medical advice; i.e., eat healthy, be active, and maintain a healthy weight. Further qualitative and quantitative research is needed to make recommendations specific to patients with breast cancer. There is also little current evidence in the Japanese population. However, a large cohort study of Japanese patients with breast cancer [15] is currently underway and the results are awaited.

## BQ1 Does consumption of alcohol increase the risk for breast cancer?

・BQ1a Premenopausal women

Statement: Alcohol consumption may increase the risk of developing breast cancer in premenopausal women.

Grade of evidence: Limited-suggestive

・BQ1b Postmenopausal women

Statement: There is a definite increased risk of developing breast cancer in postmenopausal women due to consumption of alcohol.

Grade of evidence: Convincing

## BQ2 Does smoking (including passive smoking) increase the risk for breast cancer?

Statement: It is almost certain that smoking increases the risk of developing breast cancer.

Grade of evidence: Probable

Statement: It is possible that passive smoking increases the risk of developing breast cancer.

Grade of evidence: Limited-suggestive

## BQ3 Does consumption of dairy products reduce the risk for breast cancer?

Statement: It has been suggested that intake of dairy products may reduce the risk of developing breast cancer. However, excessive intake of dairy products may increase the risk and requires caution.

Grade of evidence: Limited-suggestive

## BQ4 Does coffee consumption reduce the risk for breast cancer?

Statement: It is inconclusive whether coffee consumption decreases the risk of developing breast cancer.

Grade of evidence: Limited-no conclusion

## BQ5 Does intake of soybeans and isoflavones reduce the risk for breast cancer?

Statement: Soybean and isoflavone intake in soy foods may reduce the risk of developing breast cancer.

Grade of evidence: Limited-suggestive

## BQ6 Does taking supplements reduce the risk for breast cancer?

Statement: There is insufficient evidence that taking supplements reduces the risk of developing breast cancer.

Grade of evidence: Limited-no conclusion

## BQ7 Is obesity associated with a risk of breast cancer?

・BQ7a Premenopausal women

Statement: Obesity may increase the risk of developing breast cancer in premenopausal women.

Grade of evidence: Limited-suggestive

・BQ7b Postmenopausal women

Statement: It is certain that obesity increases the risk of developing breast cancer in postmenopausal women.

Grade of evidence: Convincing

## BQ8 Does exercise reduce the risk for breast cancer?

・BQ8a Premenopausal women

Statement: High-intensity exercise may reduce the risk of developing breast cancer in premenopausal women.

Grade of evidence: Limited-suggestive

・BQ8b Postmenopausal women

Statement: It is almost certain that exercise reduces the risk of developing breast cancer in postmenopausal women.

Grade of evidence: Probable

## BQ9 Does night work increase the risk for breast cancer?

Statement: Night work may increase the risk of developing breast cancer.

Grade of evidence: Limited-suggestive

## BQ10 Are any psychosocial factors associated with a risk for breast cancer?

Statement: No conclusion can be drawn for the associations of stress, life events, and personality tendencies with the risk of developing breast cancer.

Grade of evidence: Limited-no conclusion

## BQ11 Does radiation exposure increase the risk for breast cancer?

Statement: It is certain that exposure to high doses of radiation increases the risk of developing breast cancer, and the risk is highest for those exposed at a young age.

Grade of evidence: Convincing

Statement: It is almost certain that medical exposure such as frequent X-ray examinations and radiation therapy to the chest increase the risk of developing breast cancer, and the risk is highest for those exposed at a young age.

Grade of evidence: Probable

Statement: It is inconclusive whether low-dose exposure increases the risk of developing breast cancer.

Grade of evidence: Limited-no conclusion

## BQ12 Does benign breast disease increase the risk for breast cancer?

Statement: It is certain that proliferative lesions increase the risk of developing breast cancer. In particular, intraepithelial lesions with atypia, including atypical ductal hyperplasia, have a high risk of progressing to breast cancer.

Grade of evidence: Convincing

## BQ13 Is a family history of breast cancer a risk factor for breast cancer?

Statement: A family history of breast cancer is a definite risk factor for development of breast cancer.

Grade of evidence: Convincing

## BQ14 Does a history of diabetes mellitus increase the risk for breast cancer?

Statement: It is almost certain that a history of diabetes mellitus increases the risk of developing breast cancer.

Grade of evidence: Probable

## BQ15 Do statins reduce the risk for breast cancer?

Statement: Taking statins may not reduce the risk of developing breast cancer.

Grade of evidence: Limited-suggestive

## BQ16 Is mammographic breast density associated with a risk of breast cancer?

Statement: There is a definite increased risk of developing breast cancer with high mammographic breast density.

Grade of evidence: Convincing

## BQ17 Is it useful to administer chemopreventive drugs to reduce the risk for breast cancer?

Statement: In the current situation, in which a breast cancer risk model for Japanese women has not been established, it is not possible to conclude whether administration of drugs to prevent development of breast cancer is or is not useful.

Grade of evidence: Limited-no conclusion

## BQ18 Does breast cancer during pregnancy and lactation have a poor prognosis?

・BQ18a Pregnancy period

Statement: It cannot be concluded that breast cancer in the gestational period has a poor prognosis.

Grade of evidence: Limited-no conclusion

・BQ18b Lactation period

Statement: It is almost certain that the prognosis of breast cancer during lactation is poor.

Grade of evidence: Probable

## CQ1 Does use of low-dose oral contraceptives (OCs) or low-dose estrogen/progestin combinations (LEPs) increase the risk for breast cancer?

Statement: Use of low-dose oral contraceptives (OCs) and low-dose estrogen-progestin combinations (LEPs) may increase the risk of developing breast cancer.

Grade of evidence: Limited-suggestive

## CQ2 Does postmenopausal hormone replacement therapy (HRT) increase the risk for breast cancer?

Statement: Combined estrogen + progestin therapy using synthetic progestin in women with a uterus increases the risk of developing breast cancer when administered over a long period of time.

Grade of evidence: Probable

Statement: Estrogen monotherapy for women post-hysterectomy may also increase the risk of breast cancer.

Grade of evidence: Limited-suggestive

## CQ3 Is risk-reducing mastectomy (RRM) recommended for women with *BRCA* pathological variants?

Recommendation: Bilateral risk-reducing mastectomy (BRRM) is weakly recommended for breast cancer-naive women with *BRCA* pathologic variants.

SoR: 2, SoE: moderate, Consensus rate: 100% (38/38)

Key points: BRRM is almost certain to reduce the risk of bilateral breast cancer in breast cancer-naive patients with *BRCA* pathologic variants. On the other hand, there is much evidence that survival is affected by risk reducing salpingo-oophorectomy (RRSO), and further studies are needed to determine the effect of BRRM on survival.

Recommendation: Weak recommendation for contralateral risk-reducing mastectomy (CRRM) in patients with a diagnosis of breast cancer with a *BRCA* pathologic variant.

SoR: 2, SoE: moderate, agreement rate: 86% (32/37)

Key points: It is almost certain that CRRM reduces the risk of developing breast cancer in the contralateral breast in patients with a diagnosis of breast cancer with a *BRCA* pathologic variant. On the other hand, a meta-analysis showed a significant reduction in survival, but uncertainty remains because the effect of RRSO as a confounding factor cannot be completely ruled out. At the recommendation meeting, CRRM was given a recommendation strength of 2, taking into consideration the diversity of patient values and the remaining uncertainty regarding the effect on improved survival.

## CQ4 Is risk reducing salpingo-oophorectomy (RRSO) recommended for women with *BRCA* pathological variants?

Recommendation: Risk reducing salpingo-oophorectomy (RRSO) is strongly recommended.

SoR: 1, SoE: moderate, Consensus rate: 92% (36/39)

Key points: RRSO is certain to be effective in preventing development of ovarian and fallopian tube cancer, and prolonging overall survival. The effect of RRSO in reducing the risk for breast cancer is not yet certain, and further studies are needed. When performing RRSO, possible adverse events such as surgical menopause and the desire to have a child should be considered. RRSO should be performed based on the patient’s wishes after informed consent is obtained with provision of sufficient information before surgery.

## CQ5 Is breast-conserving therapy recommended for patients with *BRCA* pathological variants?

Recommendation: It is weakly recommended that breast-conserving therapy should not be performed in patients with breast cancer with *BRCA* pathologic variants.

SoR: 3, SoE: moderate, Consensus rate: 90% (36/40)

Key points: The rate of recurrence in the preserved breast is significantly higher in patients with *BRCA1/2* pathologic variants, and this trend becomes clearer with a longer observation period. However, no evidence of worsening survival was found. We weakly recommend that patients with breast cancer with *BRCA* pathological variants should not be treated with breast-conserving therapy unless they strongly desire this therapy.

## CQ6 Does obesity affect the prognosis of breast cancer patients?

・CQ6a Obesity at diagnosis of breast cancer

Statement:

[All breast cancer] It is certain that patients who are obese at the time of diagnosis of breast cancer have a higher risk of recurrence, breast cancer mortality, and all-cause mortality.

Grade of evidence: Convincing

Statement:

[By subtypes] The association between obesity at diagnosis of breast cancer and prognosis was examined for three breast cancer subtypes.

[Hormone receptor-positive HER2-negative breast cancer] It is almost certain that the risk of recurrence, breast cancer mortality, and all-cause mortality is higher.

Grade of evidence: Probable

[HER2-positive breast cancer] It is almost certain that the risk of recurrence, breast cancer mortality, and all-cause mortality is high.

Grade of evidence: Probable

[Triple negative breast cancer] Possible high risk of breast cancer mortality and all-cause mortality.

Grade of evidence: Limited-suggestive

・CQ6b Obesity after breast cancer diagnosis

Statement: [All breast cancer] It is almost certain that the risk of recurrence, breast cancer mortality, and all-cause mortality is higher in patients with increased obesity after breast cancer diagnosis.

Grade of evidence: Probable

## CQ7 Does dietary fat intake after initial breast cancer treatment affect prognosis?

Statement: It is inconclusive whether increased total fat intake after initial treatment for breast cancer increases the risk of recurrence.

Grade of evidence: Limited-no conclusion

## CQ8 Is maintaining a high level of physical activity recommended for patients with breast cancer?

Recommendation: It is strongly recommended to maintain a high level of physical activity after diagnosis of breast cancer.

SoR: 1, SoE: moderate, Agreement rate: 95% (37/39)

## CQ9 Is alcohol consumption associated with prognosis in breast cancer? [14]

Statement: Alcoholic beverage consumption is unlikely to increase the risk of recurrence or breast cancer mortality before or after diagnosis.

Grade of evidence: Substantial effect on risk unlikely

## CQ10 Is smoking associated with prognosis in breast cancer?

Statement: Smoking may increase the risk of recurrence in patients with breast cancer. Smoking almost certainly increases the risk of breast cancer mortality.

Grade of evidence: Probable

## CQ11 Does dietary isoflavone intake affect the prognosis of breast cancer?

Statement: Dietary intake of isoflavones may improve the prognosis of patients with breast cancer patients.

Grade of evidence: Limited-suggestive (possible)

Key points: There are no reports of an increased risk of recurrence, breast cancer mortality, or overall mortality with intake of isoflavones. A meta-analysis of three studies with breast cancer recurrence as an outcome found an overall statistically significant risk reduction. Patients with breast cancer can be recommended to consume soy because this may decrease the risk of recurrence.

## CQ12 Does consumption of dairy products affect the prognosis of breast cancer?

Statement: Dairy products are unlikely to increase the risk of recurrence, breast cancer mortality, or overall mortality in patients with breast cancer.

Grade of evidence: Limited-no conclusion

## CQ13 Are psychosocial interventions useful for patients with breast cancer?

Statements:

[Survival]: There is no evidence that psychosocial interventions prolong survival.

Grade of evidence: Limited-no conclusion

[Improvement of quality of life]: Cognitive-behavioral therapy, mindfulness, and yoga have some benefit in improving quality of life.

Grade of evidence: Limited-suggestive

[Reduction of depression]: Cognitive-behavioral therapy and mindfulness have some benefit in reducing depression.

Grade of evidence: Limited-suggestive

[Reduction of anxiety]: Cognitive-behavioral therapy, mindfulness, and yoga have some benefit in reducing anxiety.

Grade of evidence: Limited-suggestive
